# Representation of Body Orientation in Vestibular-Defective Patients Before and After Unilateral Vestibular Loss

**DOI:** 10.3389/fnsys.2021.733684

**Published:** 2021-10-27

**Authors:** Liliane Borel, Jacques Honoré, Mathilde Bachelard-Serra, Jean-Pierre Lavieille, Arnaud Saj

**Affiliations:** ^1^Cognitive Neurosciences Laboratory, UMR 7291, Aix Marseille University, CNRS, Marseille, France; ^2^SCALab, UMR 9193, University of Lille, CNRS, Lille, France; ^3^Department of Otorhinolaryngology, Head and Neck Surgery, Hôpital La Conception, APHM, Marseille, France; ^4^Department of Otorhinolaryngology, Head and Neck Surgery, CHP Clairval, Marseille, France; ^5^Laboratory for Behavioral Neurology and Imaging of Cognition, Department of Neuroscience, University of Geneva, Geneva, Switzerland; ^6^Department of Psychology, University of Montréal, Montreal, QC, Canada; ^7^Centre of Interdisciplinary Research in Rehabiliation of Montréal, CRIR/Institut Nazareth et Louis-Braille du CISSS de la Montérégie-Centre, Longueuil, QC, Canada

**Keywords:** subjective visual straight ahead, spatial orientation, unilateral vestibular loss, recovery time-course, spatial cognition

## Abstract

**Introduction:** The unilateral vestibular syndrome results in postural, oculomotor, perceptive, and cognitive symptoms. This study was designed to investigate the role of vestibular signals in body orientation representation, which remains poorly considered in vestibular patients.

**Methods:** The subjective straight ahead (SSA) was investigated using a method disentangling translation and rotation components of error. Participants were required to align a rod with their body midline in the horizontal plane. Patients with right vestibular neurotomy (RVN; *n* =8) or left vestibular neurotomy (LVN; *n* = 13) or vestibular schwannoma resection were compared with 12 healthy controls. Patients were tested the day before surgery and during the recovery period, 7 days and 2 months after the surgery.

**Results:** Before and after unilateral vestibular neurotomy, i.e., in the chronic phases, patients showed a rightward translation bias of their SSA, without rotation bias, whatever the side of the vestibular loss. However, the data show that the lower the translation error before neurotomy, the greater its increase 2 months after a total unilateral vestibular loss, therefore leading to a rightward translation of similar amplitude in the two groups of patients. In the early phase after surgery, SSA moved toward the operated side both in translation and in rotation, as typically found for biases occurring after unilateral vestibular loss, such as the subjective visual vertical (SVV) bias.

**Discussion and Conclusion:** This study gives the first description of the immediate consequences and of the recovery time course of body orientation representation after a complete unilateral vestibular loss. The overall evolution differed according to the side of the lesion with more extensive changes over time before and after left vestibular loss. It is noteworthy that representational disturbances of self-orientation were highly unusual in the chronic stage after vestibular loss and similar to those reported after hemispheric lesions causing spatial neglect, while classical ipsilesional biases were reported in the acute stage. This study strongly supports the notion that the vestibular system plays a major role in body representation processes and more broadly in spatial cognition. From a clinical point of view, SSA appeared to be a reliable indicator for the presence of a vestibular disorder.

## Introduction

Our perception of space is based on the integration of signals from vestibular, visual, and somatosensory systems. These sensory modalities allow awareness of the displacements and positions of our body and body parts, as well as the locations of objects in extra-personal space. Typically, unilateral vestibular disorders are associated with various anomalies in postural and spatial processing. Among all, this includes head and body deviations in both static (Brandt and Dieterich, 1987; Vibert et al., 1996; Borel et al., 2001) and dynamic postural conditions (Borel et al., 2002; Halmagyi et al., 2010), as well as a deviation of the locomotor trajectory (Cohen, 2000; Brandt et al., 2001; Borel et al., 2004). These postural and locomotor biases may bear witness to changes in a central representation of the verticality. The involvement of the vestibular system in the perception of the vertical has also been revealed by clinical tests (for review Brandt and Dieterich, 1994). When the injury occurs at the peripheral level, the subjective perception of verticality is deflected ipsilesionally (Friedmann, 1970; Böhmer and Mast, 1999; Vibert and Häusler, 2000; Lopez et al., 2007; Faralli et al., 2021).

The convergence of multiple sensory inputs relating to the positions and movements of the body in space within the vestibular pathways has raised questions about the implication of the vestibulocortical system to spatial cognition. In this view, the main structures of the vestibulocortical system are neither mere relays for sensory vestibular information nor mere regulatory centers for vestibulo-ocular and vestibulo-spinal reflexes. Instead, the vestibular nuclei could constitute “a preperception, premotor center for the integration of spatial information” (Lacour and Gustave Dit Duflo, 1999; Cullen, 2016), and the cortical vestibular structures seem to be a part of the neural networks underlying the internal representations of personal and extra-personal space (Bottini et al., 2001; Dieterich, 2003; Indovina et al., 2005; Lopez and Blanke, 2011).

Various studies have highlighted the consequences of vestibular loss on spatial cognition. For instance, following the unilateral vestibular loss, it has been shown that the performance of spatial memory tasks is disturbed when patients have to elaborate the representation of their own displacements in space (Péruch et al., 1999, 2005; Guidetti et al., 2007). Unilateral vestibular loss disturbed even more significantly the ability to perceive movements in a virtual environment (Péruch et al., 2005). Thus, vestibular loss modifies spatial perception even in the absence of vestibular stimulation when the subject is sitting without head movement. The unilateral vestibular loss also modifies the representation of external space. In fact, vestibular-defective patients with right-sided loss perceive a distorted, deviated space on the right side associated with compression of the contralateral hemifield (Borel et al., 2014). Interestingly, the representation of an imagined displacement is also impaired. This has been shown in mental rotation and mental scanning tasks (Grabherr et al., 2011; Péruch et al., 2011) and visuo-spatial perspective-taking tasks based on egocentric mental imagery (Deroualle et al., 2019).

Disturbances of the representation of displacements could be linked to disturbances in the representation of the orientation of the body in space. Changes in this representation have been tested in patients using subjective postural vertical tests (Bisdorff et al., 1996). The authors showed that in the acute stage after unilateral vestibular loss, patients tended to perceive their body oriented toward the lesion. Another approach to assess the perception of the body orientation, this time without body movement, consists in indicating the direction in front of oneself [subjective straight ahead (SSA)]. In healthy subjects, caloric stimulation induced a bias of the SSA to the slow phase side of the nystagmus. In the early stage after unilateral vestibular loss, the SSA was deviated to the lesion side (Hamann et al., 2009). It was then hypothesized that the side of the SSA deviation is a perceptual correlate of the gaze direction change. Then, this hypothesis was invalidated by the study of Saj et al. (2013) who described a deviation of the SSA on the side contralateral to the lesion at the compensated stage, in patients tested several years after unilateral vestibular damage who no longer showed nystagmus. Thus, it appears that the deviation of the SSA rather reflects a change in the representation of the body in space.

Such changes are consistent with the syndrome of unilateral spatial neglect classically described after a right hemisphere stroke. Neglect yields deficits in the representation of personal and extra-personal space affecting left space with the compression of the left body part and a shift of the subjective body midline toward the lesioned side (Richard et al., 2004b; Rousseaux et al., 2014). It is worth noting that in patients with unilateral peripheral vestibular loss, similar representational changes were evidenced for the first time in the study by Saj et al. (2013). These authors reported distinct SSA biases according to the side of the lesion in the chronic stage (around 2 years) after the loss. Deviations in body orientation representation occurred in patients with left vestibular loss while this representation was fairly accurate in patients with right-sided loss. The patients with a left loss presented with a contralesional shift of SSA in the horizontal plane. In contrast, in patients with unilateral peripheral vestibulopathy, no difference in SSA deviation was observed according to the side of the vestibular deficit since all patients had a rightward deviation of the SSA (Saj et al., 2021). Taken together, biases in SSA perception after vestibular loss seem to depend on the lesion side and on the postoperative time.

In this work, we conducted a longitudinal study to investigate the immediate consequences of a total unilateral vestibular loss on body orientation as well as the evolution during the recovery time course after vestibular loss. Another main issue was the differential effects depending on the side of the damage. To answer this question, we investigated the SSA of patients with right or left unilateral vestibular neurotomy or vestibular schwannoma resection. Patients were tested before surgery (*D*–1), in the early stage after surgery (*D*+7), and during the compensation stage (*D*+60). Their data were compared with those recorded the day before surgery so that each one could constitute his/her own control and analyze the possible consequences of the preoperative status on the recovery time course. The data of patients were also compared with those of healthy control subjects tested three times at the same time intervals as the patients. These data were regarded within the general framework of an asymmetry of vestibular function and in relation to the similarity of functional disorders in patients with vestibular deficits or spatial neglect.

## Methods

### Participants

Experiments were carried out on 21 patients (Tables 1, 2) before and after a right vestibular neurotomy (RVN, *n* = 8) or a left vestibular neurotomy (LVN, *n* = 13) (retrosigmoid surgery, Magnan, 2000). They suffered from a schwannoma (*n* = 17) or a severe drug-resistant Ménière's disease (*n* = 4). All the included patients were presented with a strictly unilateral vestibular pathology. Patients with motor, oculomotor, visual, or cognitive disorders were excluded from this study. None was under antivertigo medication.

**Table 1:** Demographic data of the participants.

	**Left neurotomy**	**Right neurotomy**	**Controls**
*n*	13	8	12
Age (years)	57.2 ± 11.4	51.0 ± 17.9	51.7 ± 14.5
Gender	8F, 5M	3F, 5M	7F, 5M
Dominant hand	13 right	8 right	12 right
Dominant eye	11 right, 2 left	6 right, 2 left	8 right, 4 left

*F, female; M, male*.

**Table 2:** Clinical data of patients.

	**Left neurotomy**	**Right neurotomy**	**Statistics**
**Aetiology**			
VS Grade 1	*n* = 1	*n* = 0	
VS Grade 2	*n* = 4	*n* = 4	
VS Grade 3	*n* = 6	*n* = 2	
MD	*n* = 2	*n* = 2	
**Examination at D–1**			
Delay since onset (years)	3.3 ± 3.2	2.4 ± 1.8	*p* = 0.403
Hearing loss (dB)	44.6 ± 6.9	58.6 ± 19.2	*p* = 0.959
VOR gain	0.46 ± 0.13	0.42 ± 0.16	*p* = 0.558
Spontaneous nystagmus (°/s)			
Horizontal	0.6 ± 0.8	0.5 ± 0.6	*p* = 0.870
Vertical	0.4 ± 0.7	0.5 ± 0.6	*p* = 0.636
**Examination at** ***D*****+7**			
VOR gain	0.4 ± 0.1	0.4 ± 0.1	*p* = 0.659
Spontaneous nystagmus (°/s)			
Horizontal	3.4 ± 2.7	3.6 ± 5.6	*p* = 0.936
Vertical	1.6 ± 1.3	0.7 ± 1.0	*p* = 0.146
Cyclotorsion (°)	9.8 ± 4.2	8.3 ± 4.1	*p* = 0.530
**Examination at** ***D*****+60**			
Cyclotorsion (°)	5.1 ± 4.3	5.7 ± 4.9	*p* = 0.819

*MD, Ménière's disease; VOR, vestibulo-ocular reflex; VS, vestibular schwannoma. Statistics: independent-samples t-test was used to compare the groups of patients*.

The patients were examined during three experimental sessions as follows: the day before surgery (*D*–1) and postoperatively during the acute (*D*+7) and the compensatory (*D*+60) stages. At *D*–1 and *D*+7, they had neuro-otological examinations including bithermal caloric tests, vestibulo-ocular gain, spontaneous nystagmus, torsion, and subjective visual vertical (SVV) (Table 3). The *D*–1 examination established that they exhibited a pure unilateral vestibular deficit. The patients with left and right vestibular loss did not differ in terms of their clinical status (Table 2). The performance of patients was compared with those of 12 healthy controls (CTRL) who reported to be free of sensory, motor, and cognitive disorders. None had an SVV error >±1.5°.

**Table 3:** Subjective visual vertical data of patients and controls.

	**Left neurotomy**	**Right neurotomy**	**Controls**
*D*–1	−0.1 ± 2.0	1.1 ± 1.9	0.1 ± 1.0
*D*+7	−6.9 ± 4.6	7.1 ± 5.1	0.5 ± 0.9
*D*+60	−2.7 ± 1.7	2.6 ± 1.0	0.5 ± 0.7
*p*	0.001	0.005	0.564

*Mean ± SDs, for patients, positive signs indicate contralateral deviations. For controls, positive signs indicate clockwise deviations. The p associated with the subjective visual vertical variation over time is given for each group*.

The three groups of participants did not differ (Table 1) as for age [*F*_(2,30)_ = 0.643; *p* = 0.533], gender (Yates' χ^2^ = 0.428, df = 2, and *p* = 0.807), and eye dominance (Yates' χ^2^ = 0.336, df = 2, and *p* = 0.845). All the participants were right-handed and were either emmetrope or wore corrective glasses. Each gave informed consent to this study, which was approved by the local ethics committee. The egocentric representation of space of the participants at *D*–1 has been described in a preliminary study by Saj et al. (2021).

### Materials

**Figure 1:**
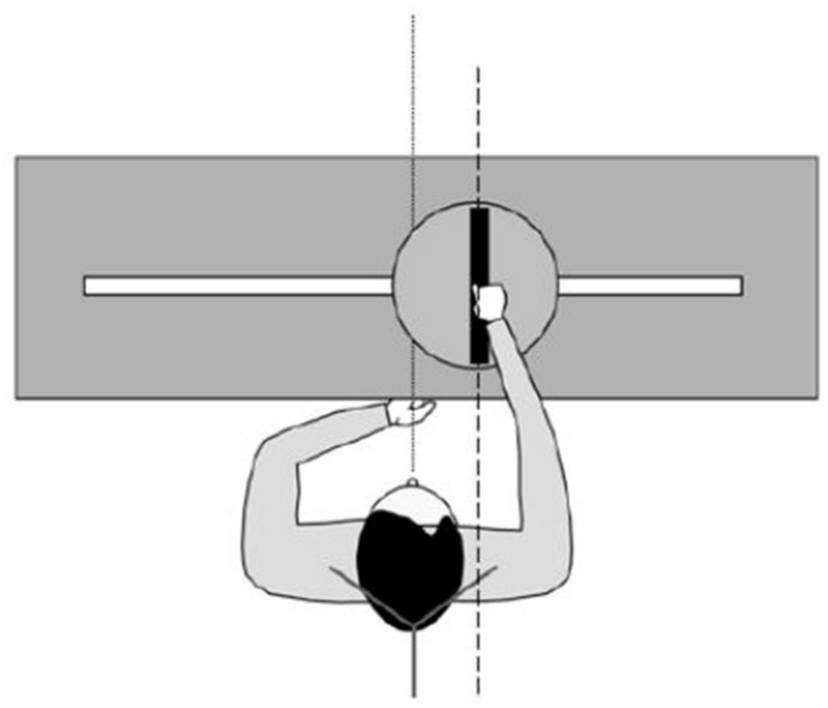
Apparatus and participant positioning for assessing the SSA.

We assessed the body midline representation disorders by means of a standard evaluation of the SSA (Richard et al., 2004b; Saj et al., 2013). The participants sat on a chair with a vertical backrest. The head was fixed by a headrest and held up in the trunk direction. Participants were facing a metal rod (25 cm long, 1.5 cm broad, and 1.5 cm thick) placed in front (50 cm) of them. The rod axis located at the navel level could rotate and slide (translation) along a 100-cm-wide slit pierced in a horizontal plate (Figure 1). A potentiometer inserted into the rod axis gave the rotation angle. The 0° position corresponded to a sagittal orientation and a positive value to a clockwise deviation of the rod. Translation movements were coded by a second potentiometer. The 0 indicated that the center of the rod was in the mid-sagittal plane of the subject, while a positive value corresponded to a rightward displacement. The accuracy of the measures was better than ±3 mm and ±0.3°. Tests were performed in darkness. Of note, 10 LEDs (2 cm long and 0.5 cm broad) were inserted in the upper side of the rod to make it visible in the darkness. Adjustments were carried out in darkness; the rod being handled at its center with the right hand. Except the rod, all parts of the apparatus were centered relative to the mid-sagittal plane of the participant.

The SSA task consisted in placing the whole rod straight ahead of the navel. The participants were instructed to imagine a line starting from the navel and extending away straight ahead of the trunk and to adjust the position of the rod in such a way that its two extremities stood on this virtual line. Depending on the trial, the rod axis was initially translated to −15 or +15 cm and rotated to −45°or +45°. The order of the four trials (one for each initial position) varied across subjects.

The participants were required to close their eyes during the setting of the location and initial tilt. Time and corrections were not limited. The trials were separated by about 20 s. The orders of initial location and initial tilt of the rod were counterbalanced across subjects.

### Statistical Analyses

In addition to the left-right convention, the data were also expressed in ipsi-contralateral coordinates, classically used to analyze the changes after unilateral vestibular loss. A minus sign indicated an ipsilesional deviation. For the SSA translation, a part of the analysis was carried out on the data signed according to left-right coordinates, a plus sign indicating a rightward error.

For each subject, the measures obtained with the different initial conditions were averaged before analysis. The rotation and translation data were analyzed separately using ANOVAs [JASP® software: Copyright 2020 University of Amsterdam (Netherlands)] with Greenhouse-Geisser corrections. The analysis design included one between-factor, i.e., the group of participants, namely, LVN, RVN, and CTRL. These ANOVAs also included the within-factor time of evaluation as follows: *D*–1, *D*+7, and *D*+60.

A detailed analysis of changes of SSA with time was obtained with orthogonal contrasts. The linear contrast (LC) was used to assess the long-term change between *D*–1 and *D*+60, and the quadratic contrast (QC) was used to assess the short-term change at *D*+7. Results were considered significant at *p* < 0.05.

## Results

**Figure 2:**
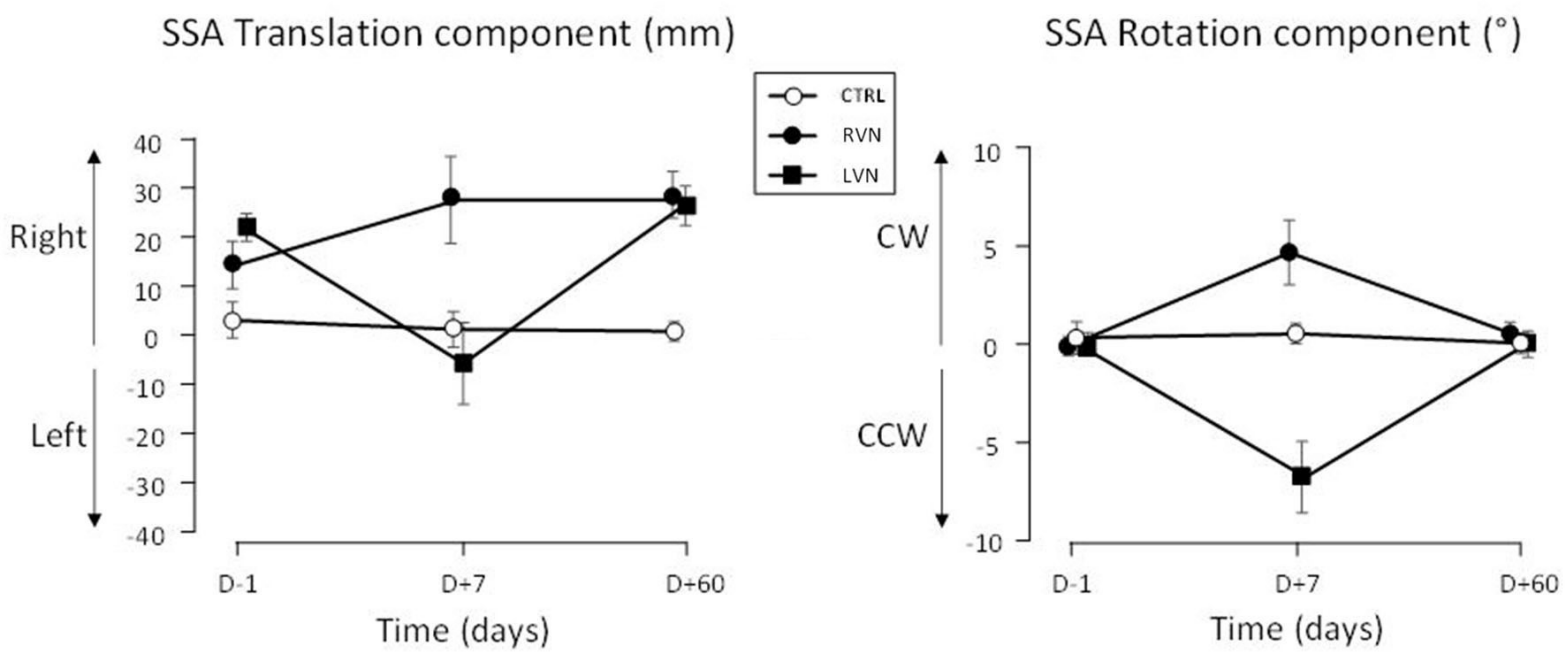
SSA deviation in translation and rotation plotted in left-right coordinates. The SSA is reported for patients with LVN (filled squares) and RVN (filled circles), and healthy controls (open circles). Positive signs indicate rightward translation and clockwise (CW) rotation. CCW: counterclockwise. The bars show the standard errors.

**Figure 3:**
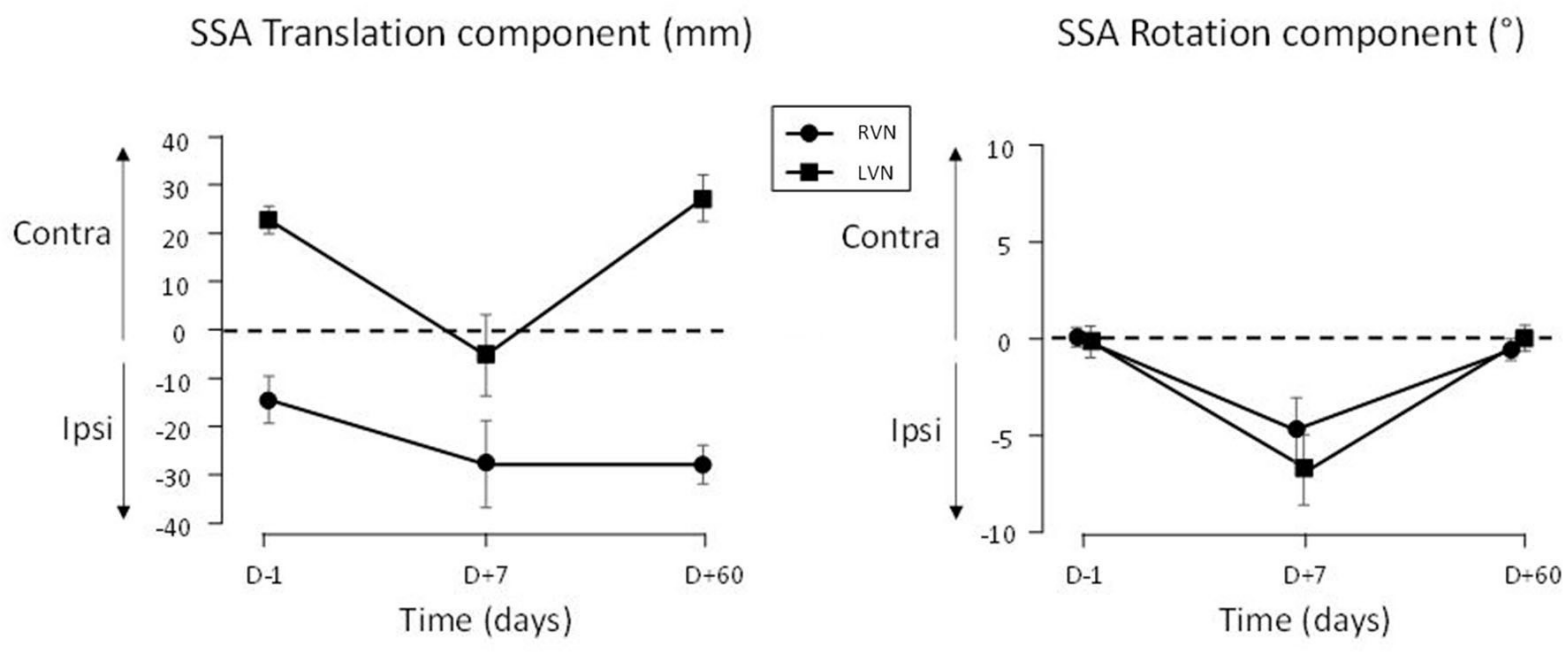
SSA deviation in translation and rotation plotted in ipsi-contra coordinates (relative to the operated side). The SSA is reported for patients with LVN (filled squares) and RVN (filled circles). Positive signs indicate contralateral translation and rotation. The bars show the standard errors.

As expected, no change occurred in SSA translation and rotation in the CTRL group. On the contrary, significant changes occurred in the patients (Figures 2, 3).

### SSA Translation

The translation deviation had two components in the groups of patients. The first component was a deviation to the right present at *D*–1 and *D*+60 in both groups (Figure 2, left panel). The initial (*D*–1) translation error correlated with the vestibular loss as evidenced by the caloric test (the greater the vestibular loss, the greater the translation, *r*_19_ = 0.46; *p* = 0.037) and the vestibulo-ocular reflex (VOR) gain (the lower the VOR gain, the greater the translation, *r*_19_ = −0.45; *p* = 0.045). However, no correlation was found with SVV, horizontal spontaneous nystagmus, or age. A second component consisted in a deviation toward the lesioned side occurring at *D*+7 (Figure 3, left panel).

**Figure 4:**
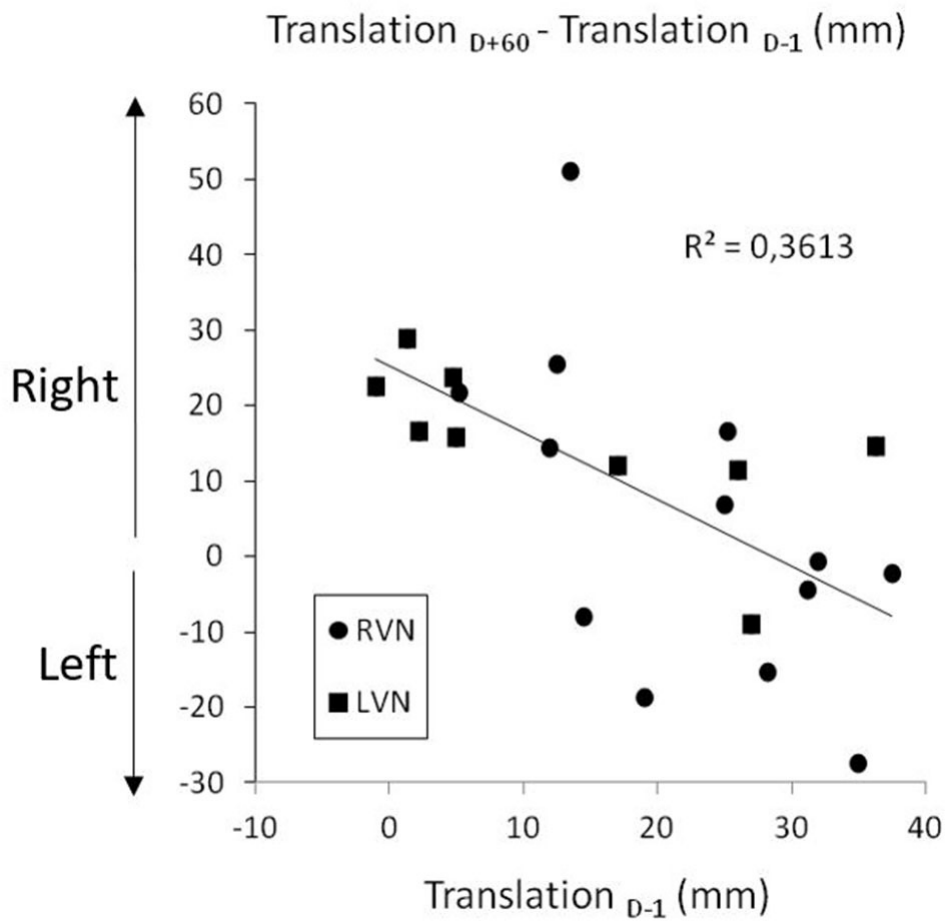
Long-term increase of the right translation bias as a function of the initial bias in right (RVN) and left (LVN) operated patients.

The SSA translation was analyzed with an ANOVA carried out on left-right coordinates (Figure 2, left panel). The time course of the deviation proved to depend on the group [*F*_(3.2, 60)_ = 6.269; *p* < 0.001; η^2^ = 0.150]. It was significant in LVN (*p* < 0.001), not in RVN (0.069) and CTRL (0.850). In the patients, the right deviation did not differ between *D*–1 and *D*+60 (LC, |*t*_60_| = 1.78; *p* = 0.080), without difference between LVN and RVN (LC, *t*_60_ = 0.885; *p* = 0.380). However, the shorter the error at *D*–1, the greater the increase between *D*–1 and *D*+60 (Figure 4; *r*_19_ = 0.567; *p* < 0.007).

The SSA translation was also analyzed on the data signed according to the lesioned side (ipsi-contra coordinates, Figure 3, left panel). The two groups of patients differed (QC, |*t*_60_| = 2.71; *p* = 0.009): the D+7 deviation was significant in LVN (QC, |*t*_60_| = 5.618; *p* < 0.001), not in RVN (QC, |*t*_60_| = 0.964; *p* = 0.339).

### SSA Rotation

The time course of the SSA rotation error was similar in both groups of patients when data were plotted in ipsi/contra coordinates (Figure 3, right panel). An ANOVA on the data expressed in left-right coordinates showed that this time course depended on the group [*F*_(2.895, 60)_ = 4.62; *p* < 0.007; η^2^ = 0.117]. It was flat in the CTRL group (*p* = 0.639), but significant in NVD (0.008) and NVL (<0.001), indicating changes during the recovery time course (Figure 2, right panel).

The rotation error of the patients was close to zero at *D*–1 and *D*+60 and did not differ between these delays (LC, |*t*_60_| = 0.232; *p* = 0.817). At *D*+7, the ipsilesional error was significant (QC, |*t*_60_| = 6.605; *p* < 0.001) and similar in both groups (QC, |*t*_60_| = 1.329; *p* = 0.189; Figure 3, right panel).

## Discussion

This study offers the first description of the immediate consequences and of the recovery time course of self-orientation representation after a complete unilateral vestibular loss. Patients with unilateral vestibular loss showed an impaired SSA direction, modulated by the side of the lesion and the postoperative time. The observed changes involved two distinct biases, namely, a translation and a rotation of SSA. They appeared simultaneously only in the early phase after the unilateral vestibular loss. Before and after unilateral vestibular neurotomy, i.e., in the chronic phases, the core impairment was a translation of the body orientation perception. In the early phase, a rotation bias appeared toward the operated side. At the same time, the translation bias was also toward the operated side, and while the bias toward the right persisted in the RVN group, it changed from the right to the left in the LVN one. More original, in the chronic stages, the translation bias was to the right for both groups of patients. As a result of these phenomena, the kinetic changes in self-orientation representation differed for patients with left and right vestibular loss.

### Long-Term Impact of Unilateral Vestibular Loss on Body Orientation Representation

The recovery time course of body orientation representation proved to be highly unusual because in both chronic periods we studied, i.e., before the neurotomy and 2 months after, the patients presented with a rightward translation whatever the side of the vestibular loss. This stands in sharp contrast with the ipsilesional biases classically found in such patients. Our data show that the consequences of the neurotomy depend on the preoperative status of the patients. Before the neurotomy, the level of the vestibulopathy differed from one patient to another, and the data showed that the greater the vestibular loss, the greater the translation error at this preoperative stage (Saj et al., 2021). In this study, from the analyses presented, we can add that the lower was the translation error before the neurotomy, the greater was its increase 2 months later when all patients had a total unilateral vestibular loss. At this stage, the body midline rightward translation was of similar amplitude in the two groups of patients.

This rightward persistent translation is reminiscent of what happens in left spatial neglect (Richard et al., 2004a,b), which occurs mostly after a right hemisphere lesion. It comforts the idea that a peripheral alteration of the vestibular system may result in neglect-like deficits, such as the impaired line bisection performance that Choi et al. (2014) observed in patients with unilateral peripheral vestibulopathy. Modulations and even transitory remissions of neglect have been obtained by stimulating (Vallar and Calzolari, 2018) or reducing vestibular input (e.g., Pizzamiglio et al., 1997). Such data have led Karnath and Dieterich (2006) to revisit the hypothesis that neglect is “a vestibular disorder.” In fact, neural networks lesioned in cases of egocentric coding deficits due to spatial neglect were shown to include the parietal lobe, the middle temporal gyrus, and the superior frontal gyrus, which receive abundant projections from the vestibular system (Rousseaux et al., 2013). The right location of the neural network lesioned in neglected patients is considered a strong argument supporting the classical right hemispheric dominance for building and representing space (Jeannerod and Biguer, 1987; Colby and Goldberg, 2001; Fasold et al., 2002). In this vein, a preliminary neuroimaging study carried out with healthy participants revealed a right hemisphere dominance for spatial representations centered on the longitudinal body axis (Saj et al., 2014). Interestingly, a right dominance for vestibular processing was also highlighted by neuroimaging explorations of right-handed healthy participants using caloric irrigation which mainly stimulates the horizontal semicircular canals (Dieterich, 2003) as well as saccular-otolith stimulations *via* the vestibular evoked myogenic potentials (Miyamoto et al., 2005; Schlindwein et al., 2008).

More recently, studies of cortical vestibular projections using diffusion tensor imaging tractography evidenced asymmetrical crossing fibers with more fibers crossing from the left vestibular nuclei to the right parieto-insular cortex (Dieterich et al., 2017). Such asymmetries, already evidenced by previous neuroimagery studies (Bottini et al., 2001; Dieterich and Brandt, 2008), could underlie asymmetrical symptoms mimicking spatial neglect, such as the rightward shift of the subjective body midline (SSA), we found in this study and the differences in neurotomy consequences according to operated side as well. Further investigations are needed to better understand how the imbalance of vestibular projections which are asymmetrically distributed between the two hemispheres results in the persisting deficits in body orientation representation.

The last point deserves attention concerning the long-term effects of vestibular loss, i.e., the different compensation courses of the SVV and SSA. This difference could have several causes. First, different vestibular cues could be involved, even if otolithic sensors are likely involved. Second, neuroimaging studies suggest that SSA and SVV involve common regions, mainly in the vestibular cortex, but also others that are different. In the SSA task, the occipital, superior parietal, and inferior frontal cortices were activated, and also in the right hemisphere, the precuneus and supplementary motor area. Activations in the insula, thalamus, and cerebellum were found in the left hemisphere (Saj et al., 2014). The SVV task activated the temporo-occipital and parieto-occipital cortical network associated with cerebellar and brainstem areas, with a right hemisphere dominance (Lopez et al., 2011; Saj et al., 2019). Although these anatomical data are not sufficient to explain the difference in compensation between SVV and SSA, they suggest that different central adaptive mechanisms could be responsible for these references that are assessed in different planes and fall under different spatial frames, i.e., geocentered and egocentered. Another possibility is that the consequences of an SSA bias could be less damaging in daily activities than those of an SVV bias in so far as many skills depend on postural abilities.

### Short-Term Impact of Unilateral Vestibular Loss on Body Orientation Representation

In the early stage after surgery, the total vestibular loss had opposite effects for left- and right-sided patients since the SSA translation bias moved ipsilesionally whatever the side of the vestibular loss. In addition, it was associated with an ipsilesional rotation bias. The SSA rotation bias, close to zero before unilateral vestibular neurotomy, returned to similar values 2 months later. Interestingly, similar changes in space representation have been described in patients with an acute unilateral peripheral vestibular deficit. These patients judged that a visual target was just in front of them when it was actually to the side of the lesion (Hamann et al., 2009).

That SSA translation and rotation biases moved toward the operated side in the early stage after surgery is consistent with what is typically observed for the visual vertical after unilateral vestibular loss and was found again in both groups of patients of this study: the SSV bias was close to zero before the neurotomy, peaked 1 week later, and strongly fell at 2 months. In fact, an imbalance of neuronal resting activity of the vestibular nuclei (Smith and Curthoys, 1988; Ris et al., 1995) was classically described as the neurophysiological basis of the static symptoms following the vestibular loss, such as the ipsilateral deviation of the SVV (Vibert and Häusler, 2000; Min et al., 2007; Borel et al., 2008). Such an asymmetry could be responsible for the transient ipsilesional deviation of the body representation observed in the days after vestibular neurotomy. At this stage, a vestibular tone imbalance in cortical areas processing vestibular signals such as posterior insula, posterolateral thalamus, and parieto-insular vestibular cortex has been described (Bense et al., 2004). Moreover, an interesting study by Becker-Bense et al. (2014) suggested that a shift of the dominant ascending input from the ipsilateral to the contralateral pathways could occur after a unilateral loss.

### Modulating Effect of the Side of the Vestibular Loss on Spatial Biases

The main result of this study is that time-course recovery of the SSA differs depending on the side of the vestibular loss, with more extensive changes over time before and after left vestibular loss. In patients with left-sided loss, the neurotomy modified the SSA translation perception to the point of reversing the sign of the deviation since in the early stage after surgery the translation error was leftward. Two months after the unilateral vestibular neurotomy, a second reversal had occurred since the body was again perceived as being translated to the right.

Only a few studies have investigated the differential consequences of vestibular loss according to the side of the disease or lesion. To our knowledge, no differential recovery after right or left vestibular loss has been described at the motor and oculomotor levels. Such effects could be restricted to high-level functions, such as spatial cognition. Different spatial performances have been reported in patients tested 2 years after unilateral vestibular neurotomy. At this stage, a bias in self-orientation perception remained for patients with a left neurotomy only (Saj et al., 2013). Similar data were reported in a study analyzing the ability to perform a visuo-spatial perspective-taking task based on egocentric mental imagery. Only patients with LVN tested 1 week after neurotomy presented an altered spatial cognitive performance (Deroualle et al., 2019). Opposite data were reported for the SVV deviation indicating a slower recovery of the SVV deviation for patients with right-sided neuritis (Toupet et al., 2014). Finally, unilateral vestibular loss led to a global impairment of the internal spatial representation, including direction and distance deficits in path integration, with similar consequences for patients with left and right vestibular loss (Péruch et al., 2005). The discordances between these studies could stem from differences in spatial tests which involves partly different central networks.

## Conclusion

This study brings two original contributions to the knowledge of the vestibular system and to the understanding of the consequences of its dysfunctions. The first contribution is a strong support to the notion that the vestibular system plays a major role in body representation processes and more broadly in spatial cognition (Lopez and Blanke, 2011; Bigelow and Agrawal, 2015). The second contribution concerns the potential interest of the representation of body orientation as a biomarker for the presence of a vestibular disorder and of the SSA as a tool in clinical practice. In fact, the permanence of the translation bias after a vestibular impairment (2 months in this study and 2 years in the study by Saj et al., 2013) appears quite remarkable. It is worth noting that the persistent alteration of the body representation could at least partly explain deficits of vestibular patients in tasks requiring a fair body-centered reference such as reaching arm movements (Raptis et al., 2007), continuous pointing toward a virtual target when the body is moved in the sagittal (Sibindi et al., 2013) or the yaw plane (Panichi et al., 2017), or performing goal-directed locomotion (Borel et al., 2004). It could also lead to alterations of the metric of personal (Saj et al., 2013) and peripersonal spaces (Borel et al., 2014), with a relative compression of one hemispace.

## Data Availability Statement

The raw data supporting the conclusions of this article will be made available by the authors, without undue reservation.

## Ethics Statement

The studies involving human participants were reviewed and approved by Comité de Protection des Personnes Sud Méditerranée I. The patients/participants provided their written informed consent to participate in this study.

## Author Contributions

LB contributed to conception, design of the work, acquisition, analysis, interpretation of data for the work, and drafting the work. JH and AS contributed to conception, design of the work, analysis, interpretation of data for the work, and drafting the work. J-PL contributed to conception of the work, analysis, interpretation of data for the work, and revising it critically. MB-S contributed to acquisition, analysis, interpretation of data for the work, and revising it critically. All authors contributed to the article and approved the submitted version.

## Funding

This work was supported by grants from CNRS, Ministère de l'Enseignement Supérieur et de la Recherche (UMR 7291).

## Conflict of Interest

The authors declare that the research was conducted in the absence of any commercial or financial relationships that could be construed as a potential conflict of interest.

## Publisher's Note

All claims expressed in this article are solely those of the authors and do not necessarily represent those of their affiliated organizations, or those of the publisher, the editors and the reviewers. Any product that may be evaluated in this article, or claim that may be made by its manufacturer, is not guaranteed or endorsed by the publisher.
